# Immunological Network Signature of Naïve Non-Oncogene-Addicted Non-Small Cell Lung Cancer Patients Treated with Anti-PD1 Therapy: A Pilot Study

**DOI:** 10.3390/cancers17060922

**Published:** 2025-03-08

**Authors:** Pasquale Sibilio, Ilaria Grazia Zizzari, Alain Gelibter, Marco Siringo, Lucrezia Tuosto, Angelica Pace, Angela Asquino, Flavio Valentino, Arianna Sabatini, Manuela Petti, Filippo Bellati, Daniele Santini, Marianna Nuti, Lorenzo Farina, Aurelia Rughetti, Chiara Napoletano

**Affiliations:** 1 Department of Computer, Control and Management Engineering, Sapienza University of Rome, 00161 Rome, Italy; pasquale.sibilio@uniroma1.it (P.S.); manuela.petti@uniroma1.it (M.P.); lorenzo.farina@uniroma1.it (L.F.); 2 Laboratory of Tumor Immunology and Cell Therapies, Department of Experimental Medicine, Sapienza University of Rome, 00161 Rome, Italy; lucrezia.tuosto@uniroma1.it (L.T.); angelica.pace@uniroma1.it (A.P.); angela.asquino@uniroma1.it (A.A.); flavio.valentino@uniroma1.it (F.V.); marianna.nuti@uniroma1.it (M.N.); aurelia.rughetti@uniroma1.it (A.R.); chiara.napoletano@uniroma1.it (C.N.); 3 Division of Oncology, Department of Radiological, Oncological and Pathological Science, Policlinico Umberto I, Sapienza University of Rome, 00161 Rome, Italy; alain.gelibter@uniroma1.it (A.G.); marco.siringo@uniroma1.it (M.S.); arianna.sabatini@uniroma1.it (A.S.); daniele.santini@uniroma1.it (D.S.); 4 Department of Medical and Surgical Sciences and Translational Medicine, Sant’Andrea University Hospital, Sapienza University of Rome, Via di Grottarossa 1035, 00189 Rome, Italy; filippo.bellati@uniroma1.it

**Keywords:** NSCLC, anti-PD1 therapy, network analysis

## Abstract

**Background/Objectives:** Non-small cell lung cancer (NSCLC) patients without gene driver mutations receive anti-PD1 treatments either as monotherapy or in combination with chemotherapy based on PD-L1 expression in tumor tissue. Anti-PD1 antibodies target various immune system components, perturbing the balance between immune cells and soluble factors. In this study, we identified the immune signatures of NSCLC patients associated with different clinical outcomes through network analysis. **Methods**: Twenty-seven metastatic NSCLC patients were assessed at baseline for the levels of circulating CD137^+^ T cells (total, CD4^+^, and CD8^+^) via cytofluorimetry, along with 14 soluble checkpoints and 20 cytokines through Luminex analysis. Hierarchical clustering and connectivity heatmaps were executed, analyzing the response to therapy (R vs. NR), performance status (PS = 0 vs. PS > 0), and overall survival (OS < 3 months vs. OS > 3 months). **Results:** The clustering of immune checkpoints revealed three groups with a significant differential proportion of six checkpoints between patients with PS = 0 and PS > 0 (*p* < 0.0001). Furthermore, significant pairwise correlations among immune factors evaluated in R were compared to the lack of significant correlations among the same immune factors in NR patients and vice versa. These comparisons were conducted for patients with PS = 0 vs. PS > 0 and OS < 3 months vs. OS > 3 months. The results indicated that NR with PS > 0 and OS ≤ 3 months exhibited an inflammatory-specific signature compared to the contrasting clinical conditions characterized by a checkpoint molecule-based network (*p* < 0.05). **Conclusions:** Identifying various connectivity immune profiles linked to response to therapy, PS, and survival in NSCLC patients represents significant findings that can optimize therapeutic choices.

## Simple Summary

This research proposes distinct immunological profiles associated with non-responding NSCLC patients who have poor survival outcomes and those with a more favorable prognosis and better performance status. An inflammatory signature characterizes the patients in the first group, while a network based on checkpoint molecules identifies NSCLC patients with better outcomes. Defining the connectivity among the molecules of each profile serves as an optimal starting point for developing combinatory targeted drugs that aim to optimize the therapeutic strategies for each patient and avoid unnecessary, toxic treatments.

## 1. Introduction

Targeting the PD1/PD-L1 pathways enhances the immune response against tumor cells, providing clinical benefits for cancer patients with advanced solid tumors, such as non-small cell lung cancer (NSCLC) [1]. Current therapies for metastatic, non-oncogene-addicted NSCLC patients are based on the tumor expression of PD-L1 (tPD-L1), which is defined by the tumor proportional score (TPS). Patients receive pembrolizumab (anti-PD1) [2] when TPS ≥ 50%, and either pembrolizumab plus chemotherapy [3,4] or a combination of nivolumab (anti-PD1) and ipilimumab (anti-CTLA4) with chemotherapy [5] when TPS <50%. The latter two treatments demonstrate comparable efficacy, with an objective response rate of 48.3%, a median progression-free survival (PFS) of 9 months, and a 4-year overall survival (OS) rate of 23.6% for pembrolizumab plus chemotherapy. In contrast, the nivolumab/ipilimumab plus chemotherapy combination shows an objective response rate of 38%, a median PFS of 6.7 months, and a 4-year OS rate of 22% [3,5]. Although tPD-L1 expression is validated and employed in clinical practice, it remains an inadequate biomarker with limited predictive value.

It is well known that anti-PD-1/PD-L1 treatments act on various components of the immune system, altering the balance among immune cells and soluble factors [6]. Identifying the connections between each factor of the immune system could help define specific signatures of patients who may benefit from immunotherapy and have a favorable prognosis, serving as valuable biomarkers for identifying patients with a defined clinical outcome.

Among immune cells, CD137^+^ lymphocytes form a T-cell subset that significantly contributes to the anti-tumor immune response. Activated CD8^+^ and CD4^+^ T cells express high levels of CD137 (4–1BB) marker, which induces effector functions, division, and survival of T cells [7,8], enhances mitochondrial metabolism in T cells [9], and promotes DNA methylation of CD8 genes [10]. These cells are recognized as tumor-specific T cells [11]. We have also demonstrated their role as predictive and prognostic biomarkers in NSCLC and other solid tumors [12,13].

Similarly, soluble checkpoints and cytokines are other critical players in the overall anti-tumor response in cancer patients. Immune cells release checkpoint molecules as alternative splice variants via microvesicles or proteolytic cleavage [14,15]. These molecules maintain their functional activity in modulating the anti-tumor immune response. Furthermore, their concentrations change during therapy, affecting the overall response rate of cancer patients [13,16,17]. Soluble PD1 (sPD1) is the most studied among patients with NSCLC. sPD1 inhibits the interaction between PD1 and PD-L1, enhancing T-cell responses, increasing the release of IFNγ, and reducing the percentage of regulatory T cells (Tregs) [18]. A similar effect has been observed for sCD80, which reverses PD-L1 signaling by binding to PD-L1 [19,20]. sPD1 is positively correlated with response and survival in NSCLC [17,21]. In contrast, high levels of sPD-L1 and sPD-L2 are associated with shorter progression-free survival and resistance to immunotherapy [21,22,23,24]. Likewise, sBTLA and sCTLA4 are considered negative regulators of the immune response linked to poor prognosis. sBTLA inhibits T-cell activation upon binding to its ligand HVEM, which is also expressed by antigen-presenting cells [25]. sCTLA4 acts as an immunosuppressive factor by blocking CD28-B7.1 ligation, inducing the release of IDO (indoleamine 2,3-deoxygenase), a tryptophan catabolic enzyme, and the FoxO3 transcription factor that regulates inflammatory cytokine production [25,26,27,28]. Recently, it was demonstrated that NK cells expressing CTLA-4 exhibited reduced cytotoxic activity, produced lower amounts of IFNγ and TNF-α, and increased IL-10 release [29].

Among the soluble factors, cytokines support the immune response toward inflammation (IL-1, IL-4, IL-6, IL-8, IL-13, IL-17, and TNFα), immune suppression (IL-10, TGF-β, and IL-35), and immune activation (IL-2, IL-12, and IFN-γ). Several cytokines, such as IFN-γ, exhibit pleiotropic activity and can function as both immune activators and suppressors [30]. Moreover, these molecules may reprogram the metabolic pathways of tumor cells, promoting metastasis and cell proliferation [31]. In recent years, serum cytokines have emerged as potential biomarkers for predicting treatment outcomes. High levels of IL-6 and IL-10 are correlated with poor survival in NSCLC patients undergoing immunotherapy [32]. Several cytokines, including IL-5, IL-6, IL-8, IL-4, and IL-10, have been identified as potential prognostic factors in NSCLC patients receiving anti-PD-1 treatment in combination with chemotherapy [33].

This evidence highlights that many immune parameters act simultaneously in the response against tumors (influencing reciprocally). The contribution of each factor strongly depends on its interaction with the immune context and tumor microenvironment that characterize each patient.

This study employs network analysis to evaluate the immunological connections among activated cells (including T-cell subsets), cytokines, and soluble immune checkpoints. These relationships were correlated with various clinical parameters, such as response to therapy, performance status, and overall survival, to identify specific immune signatures that indicate which patients are more suitable for immunotherapy.

## 2. Materials and Methods

### 2.1. Patients’ Characteristics

Twenty-seven patients diagnosed with metastatic non-oncogene-addicted NSCLC (stage IV) were enrolled at Policlinico Umberto I Hospital between 2022 and 2023. NGS analysis confirmed the mutational profile for each patient. These patients received immune checkpoint inhibitor (ICI) treatment following Italian guidelines. Patients with a TPS of ≥50% received pembrolizumab as monotherapy, while those with a TPS of <50% were treated with a combination of chemotherapy and ICIs (either pembrolizumab or nivolumab and ipilimumab), based on the physicians’ discretion. The Inclusion and Exclusion Criteria for NSCLC patients are illustrated in Supplementary Figure S1.

PS describes the patient’s level of functioning based on physical ability, daily activities, and self-care capabilities. PS = 0 indicates fully active patients with no restrictions on activities; PS = 1 characterizes patients who cannot perform strenuous activities but are able to carry out light housework and sedentary tasks; PS = 2 defines patients who can walk and manage self-care but are unable to work; PS = 3 describes patients confined to bed or a chair for more than 50% of waking hours and capable of limited self-care; PS = 4 defines patients who are completely disabled.

Each patient’s response to treatment and overall survival (OS) were evaluated. Responder (R) patients displayed a complete, partial response, or stable disease according to iRECIST criteria, whereas non-responders (NR) exhibited progression, both evaluated after 6 months of therapy. OS corresponded to the duration between the date of treatment initiation and death.

This study was conducted in accordance with good clinical practice guidelines and the Declaration of Helsinki, and it was approved by the Ethics Committee of Policlinico Umberto I (Ethical Committee Protocol, RIF.CE: 4181).

### 2.2. PBMC and Serum Collection

Peripheral blood mononuclear cells (PBMCs) and serum samples derived from 27 NSCLC patients were isolated prior to the initiation of immunotherapy. Specifically, blood samples were collected using BD Vacutainer EDTA tubes for PBMC isolation and BD Vacutainer Plus Plastic Serum tubes (both from Becton Dickinson, Franklin Lakes, NJ, USA) for serum isolation. PBMCs were stratified on Ficoll–Hypaque (Lympholite-H) (Cedarlane, Burlington, ON, Canada) and centrifuged for 30 min at 1400 rpm. The PBMCs were then collected and washed three times at 1200 rpm with PBS without Ca^2+^ and Mg^2+^ (Sigma-Aldrich, St. Louis, MO, USA). Serum samples were isolated by centrifuging the serum tubes for 30 min at 1800 rpm. PBMCs and serum were cryopreserved until use.

### 2.3. Flow Cytometry

The evaluation of T-cell subsets was conducted using cytofluorimetry with a multiparametric analysis employing the following monoclonal antibodies (MoAbs)—anti-CD3-BV510 (HIT3a clone), CD8-APC-H7 (SK1 clone), and CD137 (4–1BB)-APC (4B4–1 clone)—all sourced from BD Biosciences, San Jose, CA, USA. Live cells were identified utilizing the Live/Dead cell exclusion (Beckman Coulter, Brea, CA, USA). The negative controls were established using fluorescence minus one (FMO) and autofluorescence. All samples were processed using the DxFLEX Flow Cytometer (Beckman Coulter) and analyzed via FlowJo software (version 10.8.8, Becton Dickinson). Gating strategies are reported in the Supplementary Figure S2.

### 2.4. Cytokine and Chemokine Evaluation

Soluble immune checkpoints and cytokines were measured using the Immuno-Oncology Checkpoint 14 Plex Human ProcartaPlex Panel and the Inflammation 20 Plex Human ProcartaPlex Panel (both from ThermoFisher Scientific, Waltham, MA, USA) following the manufacturer’s instructions. The 14 immune checkpoints and 20 cytokines analyzed were the BTLA, GITR, HVEM, IDO, LAG-3, PD-1, PD-L1, PD-L2, TIM-3, CD28, CD80, CD137, CD27, and CD152 checkpoints, as well as the sE-Selectin, GM-CSF, ICAM/CD54, IFNα, IFNγ, IL1α, IL1β, IL4, IL6, IL8, IL10, IL12p70, IL13, IL17A/CTLA8, IP10/CXCL10, MCP1/CCL2, MIP1α/CCL3, MIP1β/CCL4, sP-Selectin, and TNFα cytokines. All these factors were evaluated using Luminex multiplex assays and analyzed with Bioplex Manager MP 6.2 software (Bio-Rad, Hercules, CA, USA). The instrument did not reveal the quantities of HVEM, PD-L1, GM-CSF, and MIP1α because their values were below the standard curves. The median values of these soluble factors are reported in Supplementary Tables S1 and S2.

### 2.5. Hierarchical Clustering of Circulant Molecules’ Expression Profile

The expression profiles of soluble molecules (cytokines and checkpoints) were logistically transformed. The checkpoint dataset was preprocessed by removing patients with outlier expression profiles through hierarchical clustering and applying a height threshold to the cluster dendrogram. Two patients were excluded from the analysis of checkpoints (25 patients in total) as outliers, while all patients were examined for the cytokine clustering study. 

Unsupervised hierarchical clustering was conducted in the R environment using Euclidean metrics like distance and Ward’s method. The D2 clustering algorithm was applied through the heatmap function in R. The differential expression analysis of checkpoint expression between the checkpoint-induced clusters was evaluated using the linear mixed model from the limma R package [34]. The resulting *p*-values were adjusted with the false discovery rate (FDR) to control for the expected proportion of false positives among the rejected hypotheses.

### 2.6. Differential Correlation Analysis of Multiple Clinical Conditions

Although the clustering analysis provided insights into the immune state of patients with the worst outcomes, it is limited by a small sample size. We performed a differential correlation analysis to address this limitation and provide more information about the molecular network of the immune system involved in immune therapy outcomes. In this case, the molecular profiles of patients were not evaluated in isolation; rather, the focus of the analysis shifted to all possible associations between the immune molecules and cell pairs, as well as how they change between patients with opposing therapeutic outcomes. We utilized the Differential Gene Correlation Analysis (DGCA) R package [35]. The Pearson correlation coefficient was used to assess the linear relationship between all immune molecules and cell pairs, given that a sample size of approximately 30 is generally considered acceptable when the data meet the assumptions of normality and linearity [36]. DGCA transforms sample correlation coefficients into z-scores to stabilize the variance of *r*, thereby allowing reliable comparisons of correlation coefficients across different subgroups. Then, DGCA uses the differences in z-scores to assess the statistical significance of the differentially correlated gene pairs.

In our study, we set DGCA to perform a differential correlation analysis of the immune molecule/cell pairwise Pearson’s correlation coefficients among multiple clinical conditions. The dataset, which included circulating checkpoint and membrane markers (CD3^+^CD137^+^, CD8^+^CD137^+^, CD4^+^CD137^+^ T cells), as well as cytokines, was z-normalized and used as input for DGCA. Consequently, we calculated the immune molecule/cell pairs that exhibited a differential correlation between the following comparisons: (1) non-responding vs. responding patient groups, (2) PS > 0 vs. PS = 0, and (3) OS < 12 vs. OS > 12. To identify immune molecule/cell pairs that were differentially correlated across multiple conditions, we focused on those with a difference in the absolute value of the z-scores > 1.64 (*p*-value < 0.05).

The DGCA could categorize differentially correlated immune molecule/cell pairs into nine possible categories across two conditions: +/+: positive correlation in both conditions A and B; +/0: positive correlation in condition A and no significant correlation in condition B; +/−: positive correlation in condition A and negative correlation in condition B; 0/+: no significant correlation in condition A and positive correlation in condition B; 0/0: no significant correlation in either condition; 0/−: no significant correlation in condition A and negative correlation in condition B; −/+: negative correlation in condition A and positive correlation in condition B; −/0: negative correlation in condition A and no significant correlation in condition B; −/−: negative correlation in both conditions A and B. Molecule pairs that maintain the same sign of correlation between different conditions, such as the +/+ and −/− classes, were not further investigated.

## 3. Results

### 3.1. Patients’ Characteristics

Twenty-seven patients with metastatic non-oncogenic-addicted NSCLC were enrolled in this study, as detailed in Table 1. Most histotypes were adenocarcinoma (21), with 16 patients having a TPS < 50%, while 11 had a TPS ≥ 50%. Fifteen patients were classified as PS = 0 (55%), and twelve were rated as PS > 0. Twenty patients were current or former smokers (74%), while 26% stated they had never smoked. Patients with an OS < 3 months (6 patients, 22%) were categorized as early progressors, whereas 21 exhibited an OS > 3 months. The response to treatments was assessed after six months of immunotherapy, with 14 patients considered responders (R) (52%) and 13 non-responders (NR) (48%). Median values of PFS and OS are reported in Supplementary Figure S3

### 3.2. Non-Responding Patients with PS > 0 Showed an Immunosuppressive Soluble Checkpoint Signature

**Figure 1:**
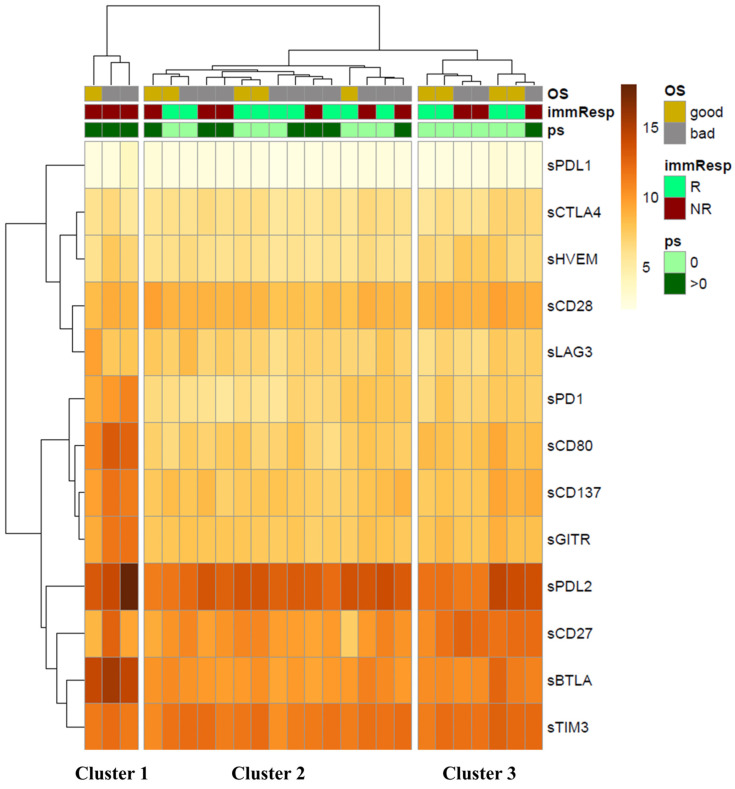
Unsupervised hierarchical clustering of the circulant checkpoints reveals three identified clusters: Cluster 1 corresponds to the left column, Cluster 2 to the central column, and Cluster 3 to the right column. The clustering includes 13 checkpoints analyzed across 25 patients (with two patients excluded due to outliers) and was conducted based on the following criteria: Overall survival (OS) categorized into good and poor (bad), with OS > 3 months indicated by a yellow square and ≤3 months by a gray square; response to treatment (immResp) after 6 months denoted as R for responders (green square) and NR for non-responders (red square); performance status (PS) = 0 indicated by a light green square and PS > 0 by a dark green square.

The unsupervised hierarchical clustering of the expression profiles of circulating checkpoints resulted in three clusters (Cluster 1: *n* = 3 (left column), Cluster 2: n = 15 (central column), Cluster 3: n = 7 (right column), Figure 1). Cluster 1 is characterized by a higher expression of six circulating checkpoints (i.e., sCD80, sBTLA, sGITR, sCD137, sPD1, and sPD-L2) compared to Clusters 2 and 3 (log2 Fold Change > 1.5, FDR < 0.05, limma modified *t*-test, Supplementary Table S1). Cluster 3 is remarkable for its significantly higher expression of sCD27 compared to Clusters 1 and 2 (log2 Fold Change > 1.5, FDR < 0.05, limma modified *t*-test, Supplementary Table S3).

Furthermore, we observe a statistically significant difference in the proportion of patients’ performance status (PS) among the clusters through the application of Fisher’s exact test (*p*-value < 0.05, Table 2). Indeed, Cluster 1 consists entirely of patients with PS > 0 who are non-responsive to therapy, whereas Cluster 2 includes 60% of responders (9/15) and 53% of patients (8/15) with PS = 0. Interestingly, 85% of non-responding patients (5/6) have a PS > 0. Cluster 3 primarily comprises patients who respond to immunotherapy (4/7, 57%) or have PS = 0 (6/7, 85.7%); notably, the only patient with PS > 0 in this cluster was also non-responsive. Moreover, no differences between the three clusters were detected when evaluating the differential proportions of the variables according to immune response and OS. All these results suggest that non-responding patients with a worse PS (Cluster 1) exhibit a more immunosuppressive profile than those with a longer response to ICIs and/or a PS = 0, confirming that poorer clinical status corresponds to a failure of the immune response.

**Figure 2:**
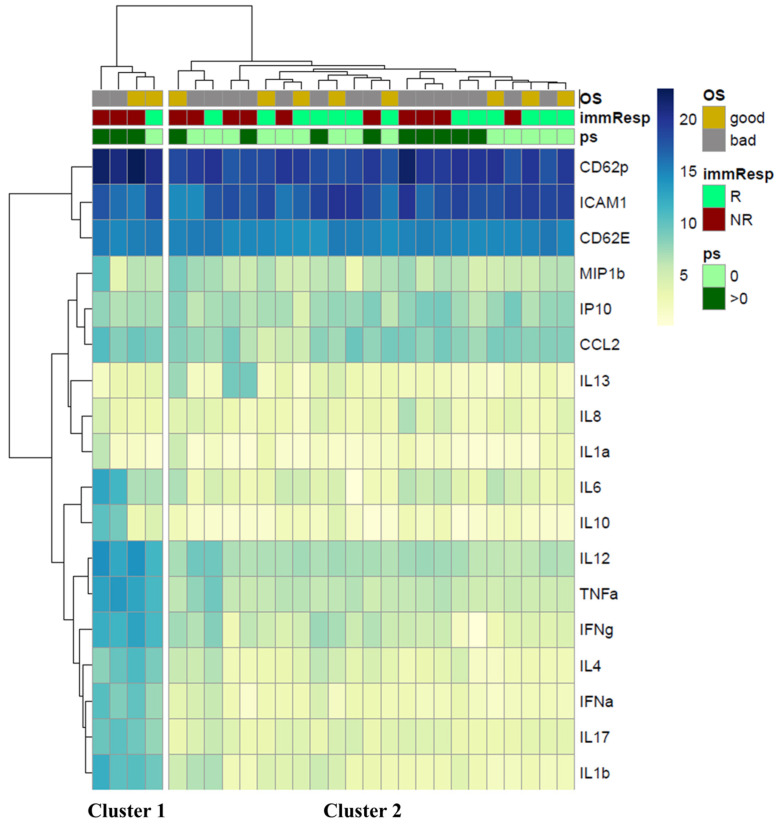
Unsupervised hierarchical clustering of the circulant cytokines. Two different clusters were identified: Cluster 1 corresponds to the left column, and Cluster 2 corresponds to the right column. The clusterization includes 18 cytokines, chemokines, and adhesion molecules analyzed in 27 patients and was performed according to 1. Overall survival (OS) divided for good and bad, corresponding to OS > 3 (yellow square) and ≤3 months (gray square), respectively; response to treatment (immResp) after 6 months (R: responders, green square; NR: non-responders, red square); performance status (PS) = 0 (light green square) and >0 (dark green square).

Although high levels of sPD1 and sCD80 are generally associated with better responses and improved survival, the increased expression of various immunosuppressive checkpoint molecules, such as sBTLA, sGITR, sCD137, and sPD-L2, shifts the immune balance toward a modified anti-tumor response. This pro-tumoral balance is further influenced by the release of multiple cytokines essential for maintaining the inflammatory environment in NR and PS > 0 patients. Indeed, unsupervised hierarchical clustering performed on the cytokines, chemokines, and adhesion molecules released into peripheral blood (Figure 2) identified two clusters. Cluster 1 (left column), consisting of 75% of NR patients with PS > 0, was primarily characterized by elevated levels of several molecules (log2 Fold Change > 1.5, FDR < 0.05, limma modified *t*-test, Supplementary Table S4), including TNFα, IL1β, IL4, IL6, IL17, and CCL2, which are associated with an inflammatory network that supports tumor growth. Cluster 2 (right column) included 56% of responders and 61% of patients classified as PS = 0. Twenty-six percent of these patients (6/23) were simultaneously classified as NR and PS > 0, indicating that proinflammatory soluble factors are not the sole parameters influencing overall response rates and patient survival.

However, no significant differences were observed between the two clusters when analyzing the differential proportions of variables based on OS, response to therapy, and PS (Table 3).

### 3.3. Non-Responding Patients Showed a Pronounced Inflammatory Network

**Figure 3:**
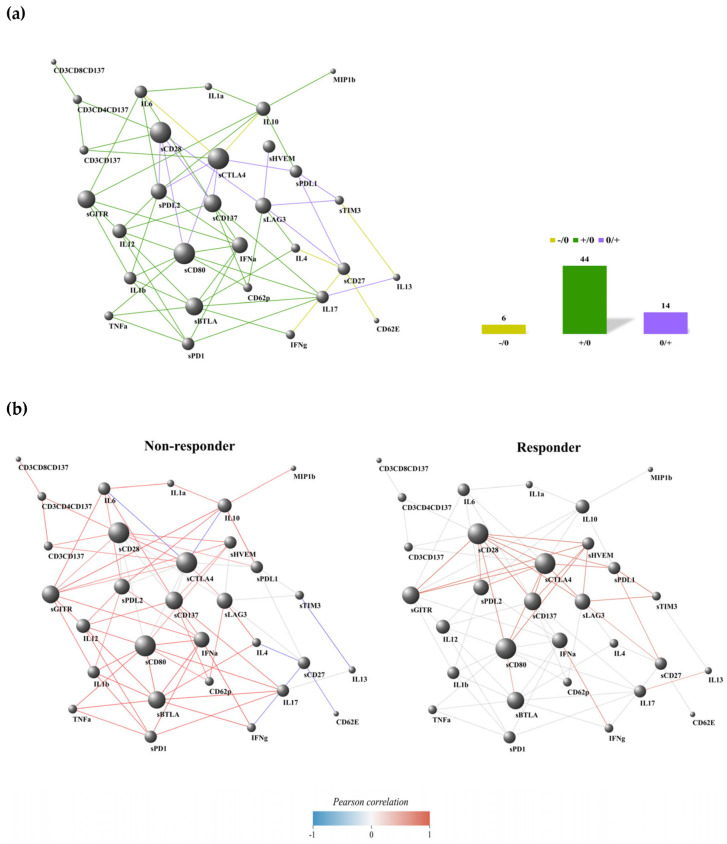
Connectivity network evaluation concerning the response to treatment in 27 naïve NSCLC patients (**a**) and dividing the analysis into non-responder and responder groups (**b**). In each network, nodes represent the immune factors (soluble cytokines/chemokines/adhesion and checkpoint molecules and CD137^+^ T-cell subsets); the link between two nodes is established when the absolute value of Spearman correlation between their expression levels is statistically significant (*p*-value ≤ 0.05). Node volume depends on the number of connections. The color of the connection depends on the class described. The yellow links and histogram identify the −/0 class that corresponds to the presence of negative correlations among the NR group, not present in the R group; the green connections and histogram discern the +/0 class that corresponds to the presence of positive correlations among the NR group, not present in the R group; the violet links and histogram are associated with the 0/+ class that corresponds to the presence of positive correlations among the R group, not present in the NR group.

The results of the DGCA analysis can be represented as a network, where the nodes are the immune soluble molecules (cytokines, chemokines, adhesion molecules, and checkpoints) and cellular subsets (CD137^+^ T cells: total, CD4^+,^ and CD8^+^). A link occurs when the molecules and cell subset pairs are differentially correlated between two conditions, according to DGCA. In the comparison between non-responding (NR) and responding (R) groups, we found 75 differentially correlated pairs of soluble molecules and cell subsets; 11 of these links were not represented because the correlation among the immune parameters showed a similar trend in both NR and R groups (NR/R: +/+ and −/−, corresponding to positive and negative correlation in both groups, respectively). Most of the differentially correlated immune molecule/cell pairs were included in the class +/0 (Figure 3a), where we observed 44 positive correlations (59%) involving diverse molecule species and lymphocyte subsets in NR, with no significant correlation in R. Similarly, other negative correlations were found to be significant in the NR group (six, accounting for 8% of the total links) compared to R, as observed in the class −/0 (Figure 3a). Conversely, 14 significant positive correlations (19%) were found in the R group compared to NR patients, as seen in class 0/+ (Figure 3a).

We highlighted the most connected nodes for each edge class (NR vs. R), specifically those with a class-specific degree above the 90th percentile of the entire class-specific degree distribution (Supplementary Table S5 ). This includes sBTLA, sCD80, IFNα, and IL12 for the +/0 class; sCD27 for the -/0 class; and sCTLA4, sCD28, and sLAG3 for the 0/+ class. In the non-responding group, there were limited negative connections, which in most cases involved a checkpoint molecule connected to one or more cytokines linked to the inflammatory pathway (IL6, IL4, IL13, and IL17) (Figure 3b). In the responding group, there were only positive connections, primarily among checkpoints, except for the interactions between IFNα and IL17-IL13. Interestingly, we found fewer connections among molecules in the responding group when comparing the network connectivity analysis between responders and non-responders. Furthermore, no common nodes were identified between the highlighted nodes within each edge class, indicating a distinct signature of network connectivity characterizing the responding and non-responding patients (Figure 3b).

### 3.4. PD-L2-IL6 and PD-L2-IL10 Connections Were Inversely Correlated in Patients with PS > 0 and PS = 0

**Figure 4:**
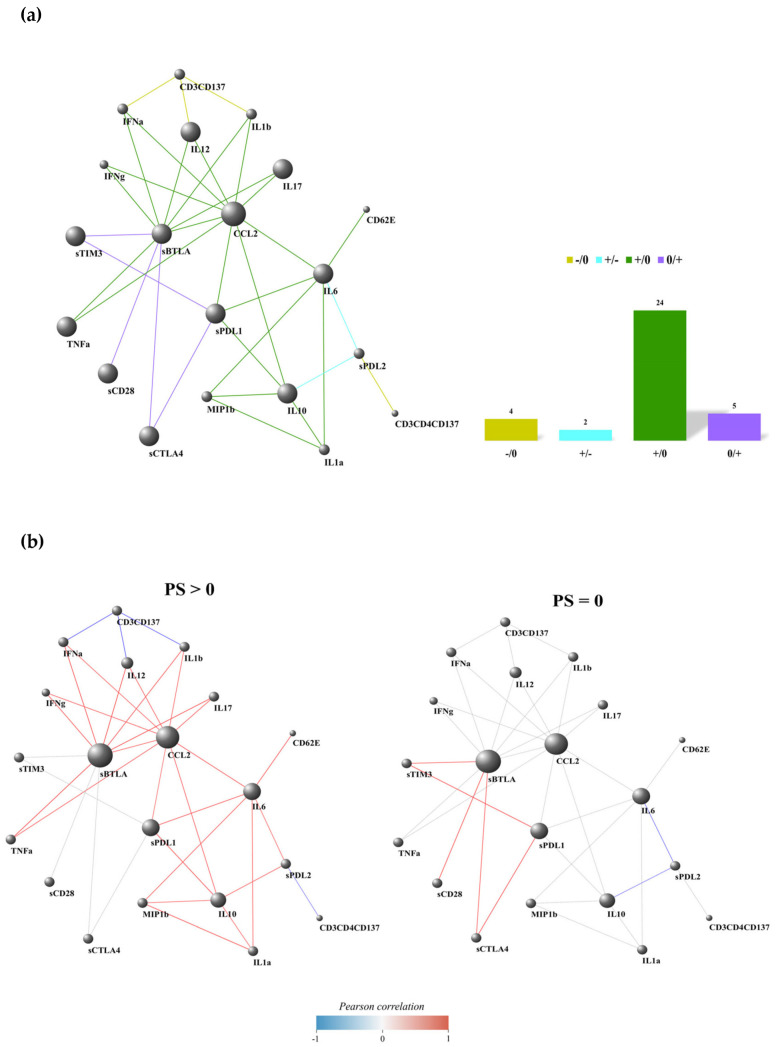
Connectivity network evaluation concerning performance status (PS) in the overall NSCLC population (**a**) and dividing the analysis into patients scored as PS > 0 and PS = 0 (**b**). In each network, nodes represent the immune factors (soluble cytokines/chemokines/adhesion and checkpoint molecules and CD137^+^ T-cell subsets); the link between two nodes is established when the absolute value of Spearman correlation between their expression levels is statistically significant (*p*-value ≤ 0.05). Node volume depends on the number of connections. The color of the connection depends on the class described. The yellow links and histogram identify the −/0 class that corresponds to the presence of negative correlations among patients with PS > 0, not present in patients with PS = 0; the sky blue links and histogram represent the +/− class that corresponds to the presence of positive and negative correlations between the PS > 0 and PS = 0 groups, respectively; the green connections and histogram represent the +/0 class that corresponds to the presence of positive correlations among the PS > 0 patients, not present in the PS = 0 group; the violet links and histogram are associated with the 0/+ class that corresponds to the presence of positive correlations among patients belonging to the PS = 0 group, not present in the patients with PS > 0.

The network analysis of performance status subgroups (PS > 0 vs. PS = 0) revealed 43 differentially correlated molecule/cellular pairs (PS > 0/PS = 0: +/0, −/0, 0/+, and +/−). Eight of these connections were excluded because a similar trend was observed between patients scored as PS > 0 and PS = 0 (+/+ and −/−). Interestingly, the two PS subgroups exhibited a similar differential correlation network between the R and NR groups (Figure 4) due to the significant overlap between the responding and PS score subgroups. Indeed, in the PS differential correlation network, most of the positive molecule pairs (24) were linked through the +/0 class (55%, Figure 4a), indicating a loss of correlation among different molecule species when transitioning from the worst state (PS > 0) to a better state (PS = 0) in the patients. A similar observation was made for the −/0 class (Figure 4a), where four negative links were noted exclusively in patients with a PS > 0 score (9%). In the 0/+ class, five significant positive correlations were identified with neighboring molecules in the PS = 0 state (12%) compared to the PS > 0 states (Figure 4a). Notably, we identified two molecule pairs (5%) that interacted through the +/− edge class (PD-L2-IL6 and PD-L2-IL10), altering the sign of their correlation when transitioning from PS > 0 to PS = 0 states (Figure 4a,b).

We highlighted the most connected nodes within each edge class of the PS differential correlation network by selecting the nodes with a class-specific degree above the 90th percentile of the entire class-specific degree distribution (Supplementary Table S6). The highlighted molecules for the +/0 class were sBTLA and CCL2, while the highlighted node for the −/0 class was the cellular subset CD3^+^CD137^+^. The selected molecule for the +/− class was sPD-L2. Notably, sBTLA emerged as the circulating checkpoint with the highest degree in the 0/+ class, playing a significant role in the transition from a PS > 0 to a PS = 0 state. Furthermore, in the PS > 0 group, the only negative connection involved the cellular subset CD137^+^ (total and CD4^+^) T cells with cytokines (IFNα, IL1β, and IL12) and PD-L2, respectively. In the PS = 0 group, we mainly observed low positive connections and two negative associations between PD-L2 and the proinflammatory cytokines IL6 and IL10 (Figure 4b).

### 3.5. Most of the PD-L1 Connections Were Interrupted in Patients with a Favorable Prognosis

**Figure 5:**
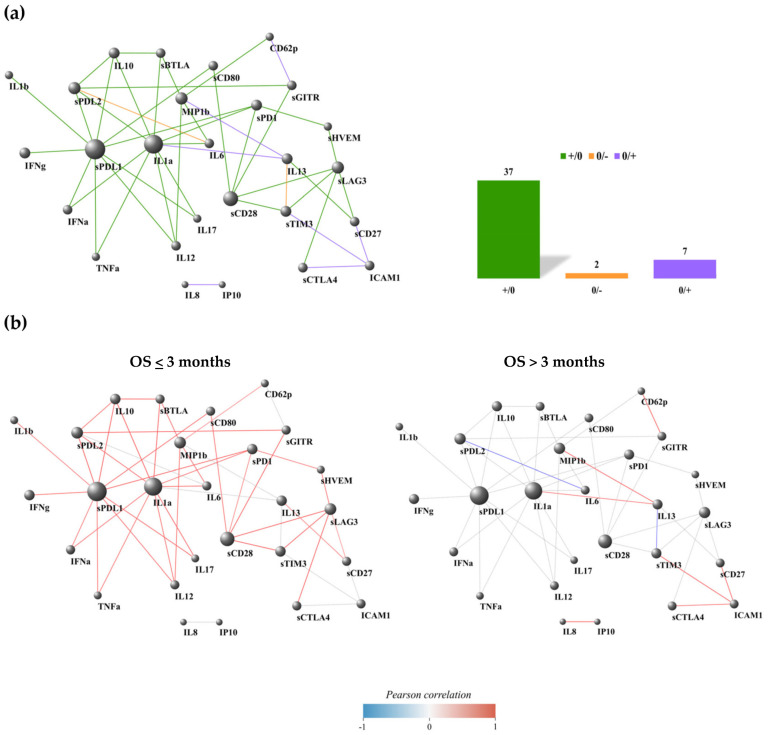
Connectivity network evaluation concerning Overall Survival (OS) in the entire NSCLC population (**a**) and dividing the analysis into patients with OS ≤ 3 months and OS > 3 months (**b**). In each network, nodes represent the immune factors (soluble cytokines/chemokines/adhesion and checkpoint molecules and CD137^+^ T-cell subsets); the link between two nodes is established when the absolute value of Spearman correlation between their expression levels is statistically significant (*p*-value ≤ 0.05). Node volume depends on the number of connections. The color of the connection depends on the class described. The green connections and histogram identify the +/0 class that corresponds to the presence of positive correlations among patients with OS ≤ 3 months, not present in patients with OS > 3 months; the orange links and histogram correspond to the presence of negative correlations among patients with OS > 3 months, not present in patients with OS ≤ 3 months; the violet links and histogram are associated with the 0/+ class that corresponds to the presence of positive correlations among patients belonging to the OS > 3 months group, not present in the patients with OS ≤ 3 months.

The analyses of DCGA were conducted by comparing patients with overall survival (OS) of less than 3 months to those with OS greater than 3 months. This timing identified patients with early progression (OS < 3 months). We found 52 differentially correlated molecule pairs (OS < 3 months/OS > 3 months: +/0, 0/−, and 0/+); 6 of these correlations were not included because the two OS groups exhibited a similar trend (+/+ and −/−). As previously described, most differentially correlated molecule pairs were found in the +/0 class (37 pairs, corresponding to 71%, Figure 5a), confirming the trend of certain molecule species, such as sPD-L1 and inflammatory cytokines like IL1β, TNFα, or IL17, being significantly correlated in the poor prognosis group (OS ≤ 3 months) and losing any significant correlation in the good prognosis group (OS > 3 months). In the 0/+ class, seven positive links were observed only in patients with OS > 3 months (13%, Figure 5a). Interestingly, we discovered two molecule pairs interacting in the 0/− class (4%, Figure 5a), represented by the nodes sTIM3-IL13 and sPD-L2-IL6.

We highlighted the most connected nodes for each edge class of the OS differential correlation network, i.e., nodes with a class-specific degree higher than the 90th percentile of the entire class-specific degree distribution (Figure 5b and Supplementary Table S7). This highlighted ICAM in the class 0/+ and sPD-L1, IL1a, sCD28, and sLAG3 for the class +/0. We only observed a positive connection for the patients in the poor survival group among the soluble factors. All positive connections were found in the group with OS > 3 months, except for sPD-L2-IL6 and IL13-TIM3, which were negatively correlated.

## 4. Discussion

The immune network is a dynamic system that remodels the tumor microenvironment. It consists of various components (cellular and soluble factors) that continuously interact, shaping the immune response and, ultimately, the clinical outcome for cancer patients. Identifying these dynamic interactions is crucial for determining specific molecular profiles that can more accurately define the complex scenario characterizing each NSCLC patient. These profiles represent selected pathways of biomarkers for describing patients with similar clinical outcomes and potential multiple immune factors for combined therapies.

In this study, we evaluated the immune network of metastatic non-oncogene-addicted NSCLC patients before the beginning of immunotherapy to identify immunological connections associated with a better response, better performance status, and longer survival (see Supplementary Table S8).

We demonstrated that non-responding patients with PS > 0 exhibited elevated serum levels of multiple soluble activating immune checkpoints (sPD1 and sCD80) and suppressive immune checkpoints (sBTLA4, sCD137, sLAG3, and sPD-L2). Despite the beneficial effects of sPD1 and sCD80 [21,37], their activity was counteracted by the presence of various immunosuppressive factors that simultaneously inhibited T-cell activation [17,22,25,38] and contributed to treatment resistance. Among these parameters, sCD137 uniquely serves as an immunosuppressor in its soluble form while acting as an immune activator when located on the plasma membrane [8]. The other parameters induce the suppression of T-cell functions in their soluble form and associate with the plasma membrane through ligand binding [39].

Moreover, the analysis of the immune network highlighted that patients who benefited from immunotherapy had optimal performance status and longer survival, and exhibited a low number of connections compared to other conditions (NR, PS > 0, and OS < 3 months). These connections were primarily positive and mainly included checkpoint inhibitors rather than cytokines. As noted in another of our analyses on various solid tumors [40], this phenomenon was particularly evident when examining the group of responding patients, where we observed interactions solely among checkpoints, except for the correlations between IFNγ-IFNα and IL17-IL13. The IFNγ pathways shared the downstream IRF1 and STAT1 molecules that bound with the promoters of PD-L1 and CXCL10, enhancing the efficacy of anti-PD1/PD-L1 inhibitors and boosting T-cell infiltration in the tumor microenvironment [41]. Additionally, IFNα exerted immunomodulatory functions on the activities of dendritic cells (DCs) and lymphocytes [42]. Simultaneously, IFNγ operated through several mechanisms, including the inhibition of angiogenesis, the suppression of proliferation, and the induction of regulatory T-cell apoptosis [43]. The roles of IL17 and IL13 were more controversial. However, their influence became more significant during tumor progression, contributing to the spread of metastasis and tumor growth in lung cancer and inducing the shift of Th1-cytokine release toward Th2, respectively [44,45]. Furthermore, the responder group displayed a distinct profile of the most connected nodes compared to the non-responders. Indeed, sBTLA, sCD80, IFNα, and IL12 were the most interacted nodes among the non-responders, while sLAG3, sCTLA4, and sCD28 characterized the responding group. These results indicated that these two patient groups were distinguished by distinct networks of molecules and pathways that defined two specific soluble signatures capable of identifying different responsiveness profiles to therapy.

The immune network excluded the CD137^+^ T-cell (total, CD8^+^, and CD4^+^) interactions in the responding group and in patients with PS = 0. These cellular subsets are identified as tumor-specific [11], and we demonstrated their role as predictive and prognostic factors in metastatic NSCLC patients [12] and in other solid tumors [46,47]. These data support the hypothesis that these cells independently exert their anti-tumor activity and are not positively associated with an inflammatory or immunosuppressive network. Conversely, in non-responders, CD137^+^ T cells are positively linked to the inhibitory molecule sCD28, and in patients with PS > 0, CD137^+^ T cells are negatively correlated with the cytokines IL1β, IL12, and IFNα. Surprisingly, in the analysis performed on OS, the nodes related to CD137^+^ T cells were absent, confirming the role of these cells as independent prognostic factors.

The DGCA’s performance, according to the PS, also indicated that the BTLA molecule was the most connected node in both the PS = 0 and PS > 0 groups. However, when we analyzed the BTLA-linked molecules in these two groups, we found that their connection profiles differed significantly. In patients with PS > 0, sBTLA was correlated with several cytokines, such as TNFα, IL1β, and IL17, contributing to a proinflammatory environment, along with sPD-L1 and sCCL2. These molecules negatively affect the cancer microenvironment [15,48]. Specifically, sPD-L1 inhibited T lymphocyte activation, while CCL2 recruited monocytes, dendritic cells (DCs), and other cells to the site of inflammation, thereby contributing to cancer pathogenesis [48]. Conversely, in patients with PS = 0, sBTLA exhibited fewer interactions and was connected only with checkpoint molecules. The connection with the sPD-L2 node was the only common link between patients with PS > 0 and PS = 0. Moreover, SPD-L2 was positively correlated with sIL6 and sIL10 cytokines in the PS > 0 group, while it was negatively associated with these cytokines in the PS = 0 group. The negative interaction between sPD-L2 and sIL6 was also observed in patients with longer overall survival (OS). These results align with the functions of these molecules. Patients with a poorer PS showed positive interactions among nodes that favored tumor growth, as they were closely correlated with the generation of a proinflammatory environment (IL6), a suppressive microenvironment (IL10), and resistance to immune checkpoints (PD-L2) [22].

The analysis of the immune network conducted based on survival revealed that the most connected node in the early progressors was sPD-L1. In the group with longer survival, all connections of sPD-L1 disappeared, except for the positive correlation with the checkpoint molecule sCD28. These two biomarkers have been proposed as negative predictive indicators of clinical response and prolonged survival in cancer patients with high levels of PD-L1 and low levels of sCD28, thereby confirming our data [49]. Furthermore, in patients with poor survival, sPD-L1 interacted with several proinflammatory and immunosuppressive cytokines, confirming that the immune connections in patients with the worst survival were also based on a proinflammatory network that supported tumor progression.

## 5. Conclusions

In conclusion, this pilot study analyzed the immunological network of several immune soluble factors and T-cell subsets that have been described as predictive and prognostic factors when evaluated individually, aiming to understand the complex immunological scenario of each NSCLC patient. We proposed several immune profiles to identify responding patients and those with longer survival, which could be utilized to optimize personalized therapeutic strategies. Despite the limited number of patients, it was evident that non-responding patients with poor clinical status and survival exhibited an inflammatory-specific signature, which was switched off in patients who responded to therapy and had improved performance status. Patients with a more favorable prognosis were characterized by a network based on checkpoint molecules, likely less conducive to promoting tumor growth. This study reported significant findings in the field of precision medicine, where identifying immune profiles and their connectivity represents a new challenge for further investigation.

## Figures and Tables

**Table 1:** Patients’ characteristics.

Tot	N°27 (100%)
Sex	
Male	18 (67%)
Female	9 (33%)
AgeMedian range	48–84
≤75	22 (81)
>75	5 (19)
Histotype	
Squamous	6 (22)
Adenocarcinoma	21 (78)
TPS	
<50%	16 (59)
≥50%	11 (41)
EOCG Performance Status	
0	15 (55)
>0	12 (45)
Overall Survival	
≤3 months	6 (22)
>3 months	21 (78)
Smoking Status	
Current	13 (48)
Former	7 (26)
Non-smoker	7 (26)
Response to immunotherapy	
Yes	14 (52)
No	13 (48)

**Table 2:** The differential proportions of patients’ overall survival, response to ICIs, and performance status evaluated in the soluble checkpoint inhibitor clustering.

Clinical Variables		Cluster 1	Cluster 2	Cluster 3	*p*-Value Fisher Test
Overall survival	Good	2	10	3	NS
	Bad	1	5	4	
Response to therapy	R	3	6	3	NS
	NR	0	9	4	
Performance Status	0	0	8	6	*p* < 0.0005
	>0	3	7	1	

NS: not statistically significant.

**Table 3:** The differential proportions of patients’ overall survival, response to ICIs, and performance status evaluated in the cytokine/chemokine clustering.

Clinical Variables		Cluster 1	Cluster 2	*p*-Value Fisher Test
Overall Survival	Good	15	2	NS
	Bad	8	2	
Response to therapy	R	10	3	NS
	NR	13	1	
Performance Status	0	3	1	NS
	>0	9	14	

NS: not statistically significant.

## Data Availability

Data were generated by the authors and are included in the article.

## Supplementary Materials

The following supporting information can be downloaded at https://www.mdpi.com/article/10.3390/cancers17060922/s1, Figure S1: Schematic representation of inclusion and exclusion criteria during NSCLC enrollment Figure S2: Cytofluorimetric analysis of CD137 T subsets; Figure S3: Kaplan Meier curves of Progression-Free Survival (PFS) and Overall Survival (OS) of NSCLC patients; Table S1: Median values and SEM of Cytokine and Chemokines/Adhesion molecules evaluated in 27 NSCLC patients; Table S2: Median values and SEM of checkpoint molecules evaluated in 27 NSCLC patients; Table S3: Differential expression analysis of circulating checkpoints induced clusters; Table S4: Differential expression analysis of circulating cytokines induced clusters; Table S5: Differential correlation analysis between NR vs R patients; Table S6: Differential correlation analysis between Performance Status (PS) > 0 vs PS = 0 patients; Table S7: Differential correlation analysis between Overal Survival (OS) < 3 months vs OS > 3 months; Table S8: Summury of the main results.

## Abbreviations

The following abbreviations are used in this manuscript:

NSCLC	Non-small cell lung cancer
PS	Performance status
OS	Overall survival
TPS	Tumor proportional score
IDO	Indoleamine 2,3-deoxygenase
FoxO3	Forkhead Box O3
EOCG	Eastern Cooperative Oncology Group
FDR	False discovery rate
DGCA	Different gene correlation
