# Supplementary material for: Immunological Network Signature of Naïve Non-Oncogene-Addicted Non-Small Cell Lung Cancer Patients Treated with Anti-PD1 Therapy: A Pilot Study
Source: Cancers (Basel). 2025 Mar 8;17(6):922. doi: 10.3390/cancers17060922 (PMC11939851; doi:10.3390/cancers17060922)
Supplement: Supplementary file 1 [file cancers-17-00922-s001.zip › Supplementary Figures.pptx]

## Slide 1
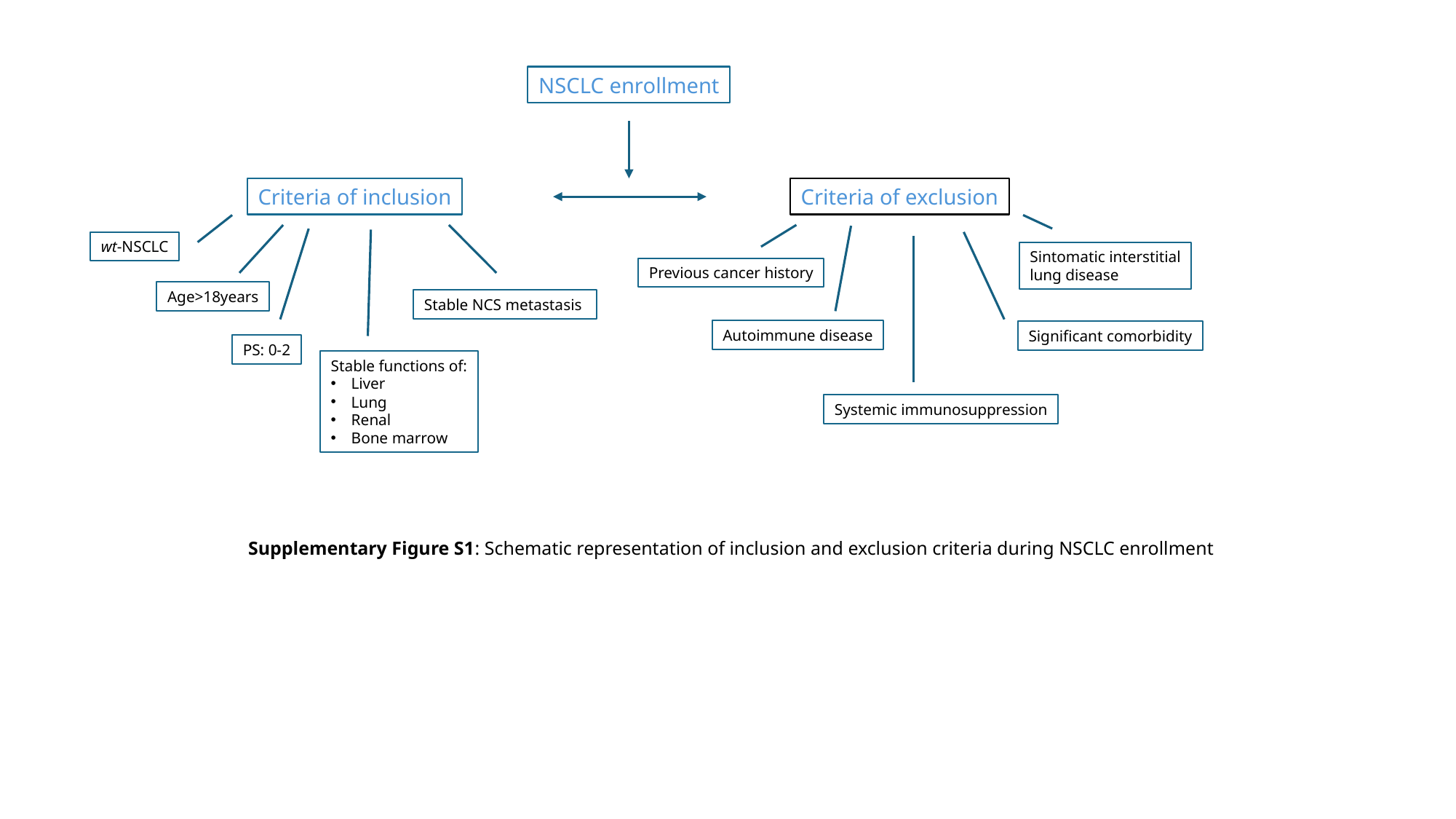

NSCLC enrollment
Criteria of inclusion
Criteria of exclusion
wt-NSCLC
Sintomatic interstitial
lung disease
Previous cancer history
Age>18years
Stable NCS metastasis
Autoimmune disease
Significant comorbidity
PS: 0-2
Stable functions of:
Liver
Lung
Renal
Bone marrow
Systemic immunosuppression
Supplementary Figure S1: Schematic representation of inclusion and exclusion criteria during NSCLC enrollment

## Slide 2
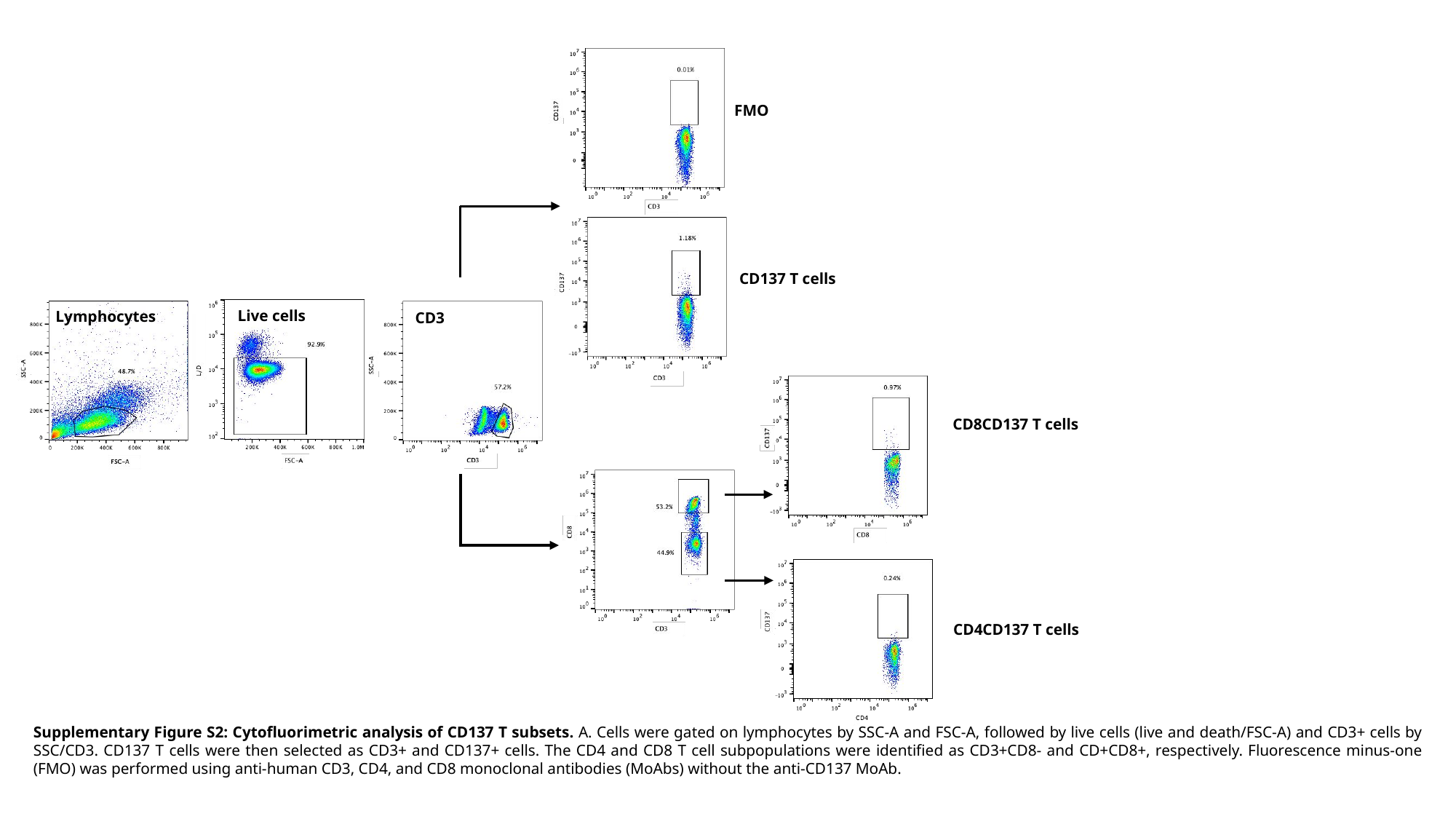

FMO
CD137 T cells
Live cells
Lymphocytes
CD3
CD8CD137 T cells
CD4CD137 T cells
Supplementary Figure S2: Cytofluorimetric analysis of CD137 T subsets. A. Cells were gated on lymphocytes by SSC-A and FSC-A, followed by live cells (live and death/FSC-A) and CD3+ cells by SSC/CD3. CD137 T cells were then selected as CD3+ and CD137+ cells. The CD4 and CD8 T cell subpopulations were identified as CD3+CD8- and CD+CD8+, respectively. Fluorescence minus-one (FMO) was performed using anti-human CD3, CD4, and CD8 monoclonal antibodies (MoAbs) without the anti-CD137 MoAb.

## Slide 3
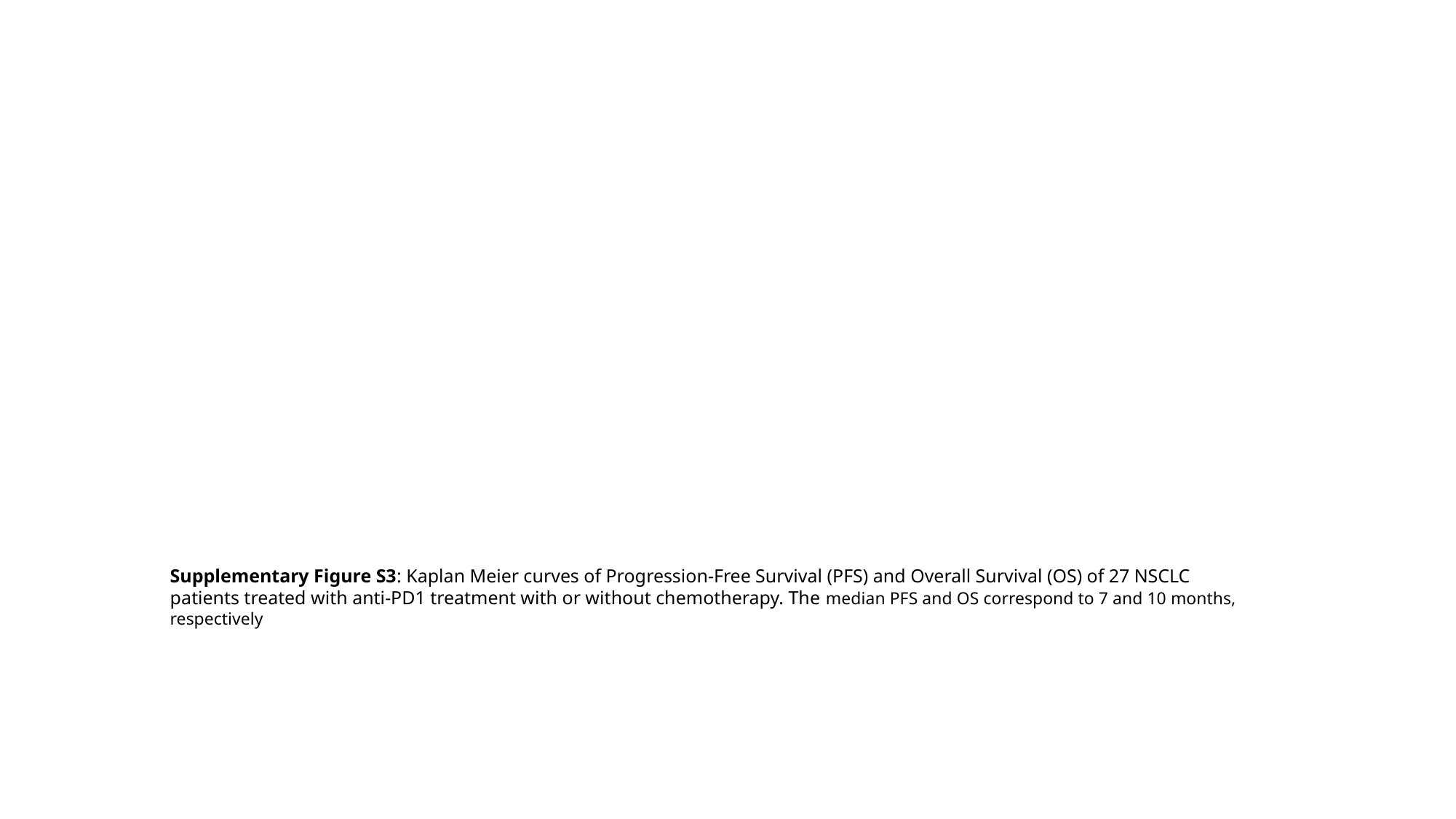

Supplementary Figure S3: Kaplan Meier curves of Progression-Free Survival (PFS) and Overall Survival (OS) of 27 NSCLC patients treated with anti-PD1 treatment with or without chemotherapy. The median PFS and OS correspond to 7 and 10 months, respectively
